# Bibliography

**Published:** 1891-11

**Authors:** 


### BIBLIOGRAPHY.

Chemistry of the Carbon Compounds, or Organic Chemistry. By Professor Victor von Richter, University of Breslau. Translated by Edgar F. Smith, Professor of Chemistry, University of Pennsylvania. Philadelphia: P. Blakiston, Son & Co., 1891.

The appearance of a second edition of this most excellent work so soon after the appearance of the first edition is a sure indication of the esteem in which this book is held by chemists and students. The second edition, which is a translation from advanced sheets of the sixth German edition furnished by the courtesy of Professor von Richter himself, is a volume of 1040 pages, an increase of about 300 pages over the first edition, and is a most complete compendium of organic chemistry brought up to the very latest date.

All of the approved methods of elementary organic analysis are described in detail, together with the methods of determining molecular formulas, including the recent methods of Van't Hoff and of Raoult.

The most modern views relating to the theory of the structure of organic compounds, including the stereo-chemical and tautomeric theories, are admirably explained.

The special part descriptive of the properties of organic compounds is complete and brought up to the present time.

The English-reading chemical world certainly owes a debt of gratitude to Professor Smith for rendering available this most excellent work, which serves not only as one of the best text-books and a complete work of reference, but also to a great extent as a guide in prosecuting work in organic chemistry in the laboratory.

M.

BOOKS RECEIVED.

The following books have been received. Notices will appear as rapidly as possible :

"Annual of the Universal Medical Sciences." Edited by Charles E. Sajous, M.D. In five volumes. F. A. Davis, publisher, Philadelphia.

"A Short Manual of Analytical Chemistry." By John Muter, M.A., Ph.D., etc. First American from the fourth English edition. P. Blakiston, Son & Co., Philadelphia, 1891.

"The Pocket Materia Medica and Therapeutics." By C. Henri Leonard, A.M., M.D. Illustrated Medical Journal Co., Detroit, Mich., 1891.

"The Vest-Pocket Anatomist." (Founded upon Gray.) Fourteenth revised edition. Illustrated Medical Journal Co., Detroit.

